# HTLV-1 CTCF-binding site is dispensable for in vitro immortalization and persistent infection in vivo

**DOI:** 10.1186/s12977-019-0507-9

**Published:** 2019-12-21

**Authors:** Michael P. Martinez, Xiaogang Cheng, Ancy Joseph, Jacob Al-Saleem, Amanda R. Panfil, Marilly Palettas, Wessel P. Dirksen, Lee Ratner, Patrick L. Green

**Affiliations:** 1 Center for Retrovirus Research, The Ohio State University, Columbus, OH USA; 2 Department of Veterinary Biosciences, The Ohio State University, Columbus, OH USA; 3 Center for Biostatistics, The Ohio State University, Columbus, OH USA; 4 Comprehensive Cancer Center, The Ohio State University, Columbus, OH USA; 5 Division of Oncology, Washington University, St. Louis, MO USA

**Keywords:** HTLV-1, CTCF, Retrovirus, Immortalization, Persistence

## Abstract

**Background:**

Human T-cell leukemia virus type 1 (HTLV-1) is the etiologic agent of adult T-cell leukemia/lymphoma (ATL) and the neurological disorder HTLV-1-associated myelopathy/tropical spastic paraparesis (HAM/TSP). The exact mechanism(s) through which latency and disease progression are regulated are not fully understood. CCCTC-binding factor (CTCF) is an 11-zinc finger, sequence-specific, DNA-binding protein with thousands of binding sites throughout mammalian genomes. CTCF has been shown to play a role in organization of higher-order chromatin structure, gene expression, genomic imprinting, and serve as a barrier to epigenetic modification. A viral CTCF-binding site (vCTCF-BS) was previously identified within the overlapping *p12* (sense) and *Hbz* (antisense) genes of the HTLV-1 genome. Thus, upon integration, HTLV-1 randomly inserts a vCTCF-BS into the host genome. vCTCF-BS studies to date have focused primarily on HTLV-1 chronically infected or tumor-derived cell lines. In these studies, HTLV-1 was shown to alter the structure and transcription of the surrounding host chromatin through the newly inserted vCTCF-BS. However, the effects of CTCF binding in the early stages of HTLV-1 infection remains unexplored. This study examines the effects of the vCTCF-BS on HTLV-1-induced in vitro immortalization and in vivo viral persistence in infected rabbits.

**Results:**

HTLV-1 and HTLV-1∆CTCF LTR-transactivation, viral particle production, and immortalization capacity were comparable in vitro. The total lymphocyte count, proviral load, and *Hbz* gene expression were not significantly different between HTLV-1 and HTLV-1∆CTCF-infected rabbits throughout a 12 week study. However, HTLV-1∆CTCF-infected rabbits displayed a significantly decreased HTLV-1-specific antibody response compared to HTLV-1-infected rabbits.

**Conclusions:**

Mutation of the HTLV-1 vCTCF-BS does not significantly alter T-lymphocyte transformation capacity or early in vivo virus persistence, but results in a decreased HTLV-1-specific antibody response during early infection in rabbits. Ultimately, understanding epigenetic regulation of HTLV-1 gene expression and pathogenesis could provide meaningful insights into mechanisms of immune evasion and novel therapeutic targets.

## Background

Human T-cell leukemia virus type 1 (HTLV-1) is the first discovered human retrovirus with an estimated 5–10 million individuals infected worldwide [1, 2]. HTLV-1 is the etiologic agent of a non-Hodgkin’s peripheral T-cell malignancy called adult T-cell leukemia/lymphoma (ATL) and a demyelinating lymphocytic meningomyelitis termed HTLV-1-associated myelopathy/tropical spastic paraparesis (HAM/TSP) [3]. Approximately 5–10% of infected individuals will develop ATL or HAM/TSP after a prolonged period of clinical latency [4, 5]. It remains poorly understood why some infected individuals develop HTLV-1 associated disease while others do not.

Recently, a CCCTC-binding factor (CTCF) binding site was identified within the HTLV-1 provirus [6]. CTCF is a multifunctional, 11-zinc-finger, DNA-binding protein with tens of thousands of binding sites throughout mammalian genomes [7, 8]. CTCF has been shown to play a role in higher-order chromatin structure, gene expression, genomic imprinting, and serve as a barrier to epigenetic modification [9–11]. Additionally, several tumorigenic viruses including Kaposi’s sarcoma-associated herpesvirus, human papillomavirus, and Epstein-Barr virus have been found to utilize CTCF to regulate differential viral gene expression [12].

Unlike HIV-1, HTLV-1 integration appears to have no strong integration site predilection [13, 14]. Thus, HTLV-1 integration randomly inserts a viral CTCF-binding site (vCTCF-BS) into the host genome. CTCF has been shown to bind the HTLV-1 genome and alter the structure and transcription of the surrounding host chromatin [6, 15]. How these interactions affect HTLV-1 pathobiology during early infection warrants investigation and is the focus of this study.

High HTLV-1 proviral load in asymptomatic carriers is recognized as a risk factor for the development of both ATL and HAM/TSP [16, 17]. Thus, factors that contribute to alterations in proviral load are of significant importance to HTLV-1 pathogenesis. Two such viral factors are HTLV-1 trans-activator from the X region (*Tax*) and HTLV-1 basic leucine zipper factor (*Hbz*). *Tax* is considered the primary oncogene of HTLV-1. Tax drives proviral transcription via transactivation of the 5′ HTLV-1 long terminal repeat (LTR) and has been shown to promote cellular proliferation via dysregulation of multiple pathways including activation of NF-κB and cyclin dependent kinases 2/4 [18]. Hbz has been shown to negatively regulate Tax and independently stimulate cell proliferation in both its protein and RNA forms [18]. It has been shown that Tax and Hbz play a critical role in viral persistence using an established animal model of HTLV-1 infection, the New Zealand White (NZW) rabbit [19, 20]. Changes in host or proviral gene expression via abnormal chromatin looping as a result of ectopic insertion of a vCTCF-BS into the host genome could result in altered persistence during early infection.

The aims of this study are to determine the effects of vCTCF-BS ablation on in vitro immortalization capacity via a co-cultivation assay and in vivo persistence, utilizing the NZW rabbit as a model for early infection. Our results indicate that abrogation of CTCF binding to the vCTCF-BS does not alter in vitro immortalization capacity or in vivo persistence, but does significantly decrease the in vivo HTLV-1-specific antibody response when compared to appropriate HTLV-1 controls.

## Results

### Construction and characterization of HTLV-1 proviral clones

**Fig. 1:**
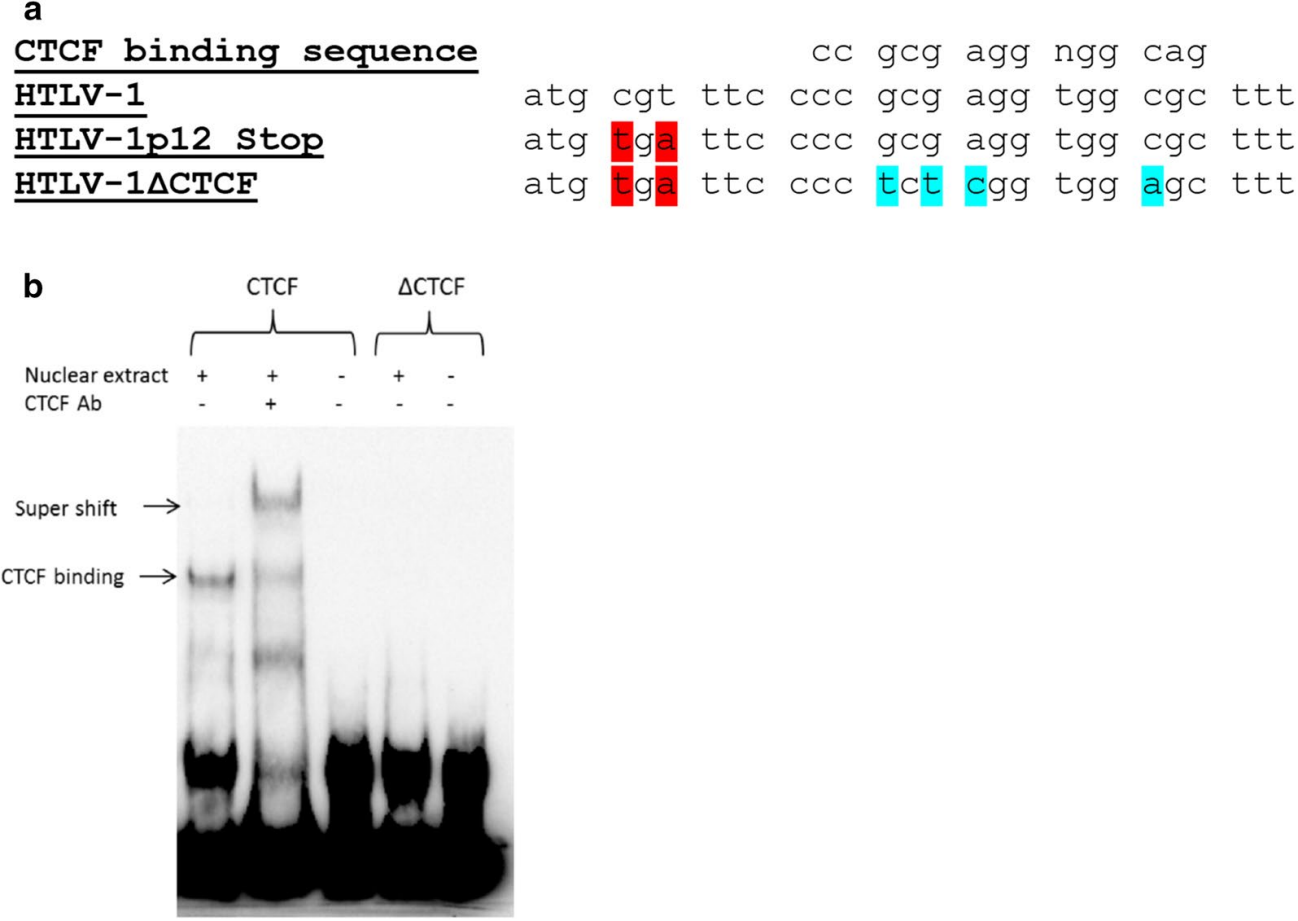
Construction of HTLV-1 proviral clones. Site-directed mutagenesis was utilized to abrogate CTCF binding. **a** Alignment of the consensus CTCF-binding sequence with HTLV-1, HTLV-1p12Stop, and HTLV-1∆CTCF in the context of accessory gene *p12.* HTLV-1∆CTCF contains mutations that abolish CTCF binding (blue). These mutations disrupt the *p12* reading frame, therefore a stop mutation (red) that truncates p12 by 23 amino acids was introduced immediately upstream. HTLV-1p12Stop serves as a control by containing only the aforementioned stop codon (red). **b** Abolition of CTCF binding was confirmed via electrophoretic mobility shift assay. EMSA was performed using the Light Chemiluminescent EMSA kit (Thermo Scientific) and following the manufacturer’s protocol with some modification. Briefly, nuclear extract of 293T cells transfected with the plasmid overexpressing human CTCF protein was incubated with biotin labeled target DNA in the presence or absence of the CTCF antibody. Protein bound DNA was separated from unbound DNA in a polyacrylamide gel and transferred to a nylon membrane. DNA was then cross-linked to the membrane. The membrane was incubated with streptavidin–horseradish peroxidase conjugate in blocking buffer and then exposed to the substrate solution. Biotin labeled DNA was detected by using Chemidoc XRS + molecular imager (Bio-Rad)

**Fig. 2:**
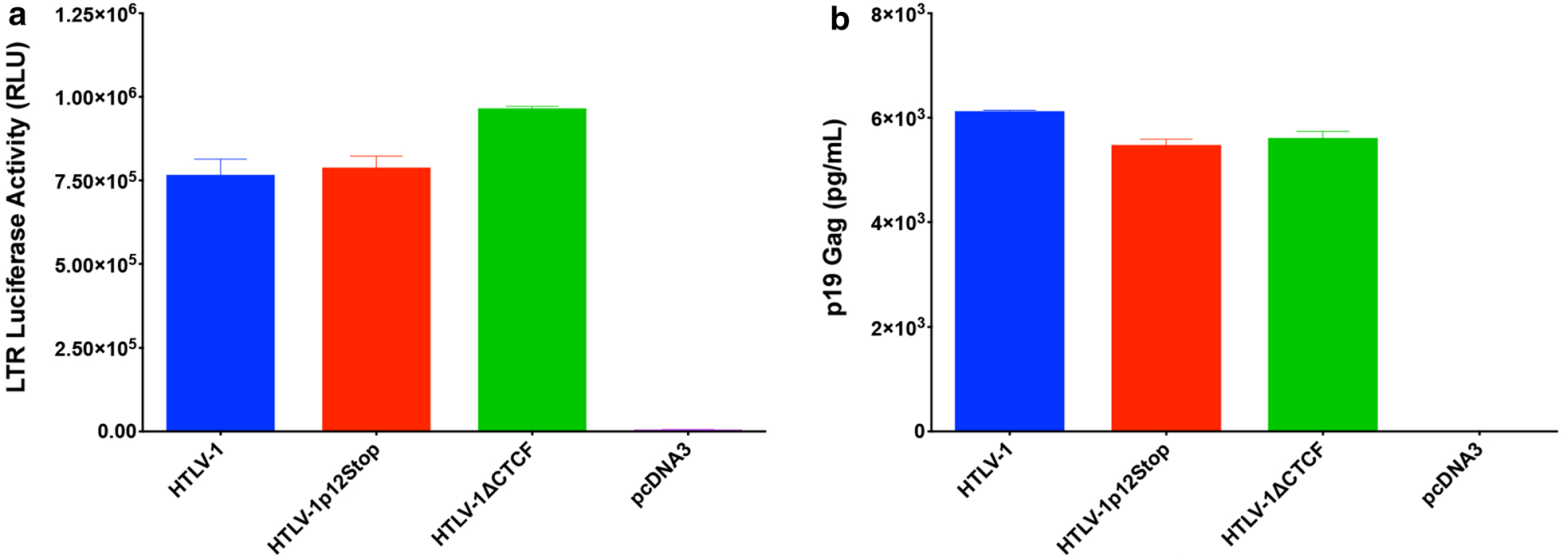
Characterization of HTLV-1 proviral clones. An HTLV-1 LTR-luciferase assay and ELISA specific for HTLV-1 structural protein p19 Gag were performed as in vitro functional assays. HEK293T cells were co-transfected with an LTR-1-Luc reporter construct and 1 µg of HTLV-1, HTLV-1∆CTCF, HTLV-1p12Stop, or empty (pcDNA3) plasmids. 48 h post-transfection cells and supernatant were collected for LTR-transactivation luciferase assay **a** and p19 Gag ELISA **b**, respectively. HTLV-1, HTLV-1∆CTCF, HTLV-1p12Stop and proviral clones generated comparable LTR-transactivation and p19 Gag production

In order to determine the role of the vCTCF-BS in HTLV-1-mediated cellular immortalization in vitro and viral persistence in vivo, we generated two mutant proviral clones using the well-characterized HTLV-1 molecular clone ACH (HTLV-1). HTLV-1∆CTCF contains several point mutations within the consensus vCTCF-BS while avoiding introduction of mutations to the opposite-strand coding sequence of the *Hbz* gene. However, the vCTCF-BS mutations do result in changes to *p12*, a sense transcribed HTLV-1 accessory gene. Previous reports have shown that mutant ablation of p12 (ATG changed to GTG) had no measurable effect on HTLV-1 transforming capacity of primary human T-cells in cell culture nor infectivity and persistence in inoculated rabbits [21]. However, in lieu of producing a *p12* gene product with multiple substitutions and potentially confounding results, an additional mutation was introduced in *p12*, immediately upstream of the vCTCF-BS mutations. This mutation results in deletion of the carboxy terminal 23 amino acids of p12 (Fig. 1a). The HTLV-1p12Stop contains only the p12 stop point mutations and thus will serve as an additional control accounting for the p12 deletion in our viral studies. Using an electrophoretic mobility shift assay (EMSA), Fig. 1b shows that the ∆CTCF sequence in HTLV-1∆CTCF fails to bind CTCF compared to the wild-type HTLV-1 sequence. We next determined whether HTLV-1∆CTCF or HTLV-1p12Stop mutant proviruses had altered Tax-mediated LTR gene expression. Cotransfection of either HTLV-1 or mutant HTLV-1 proviral clones, as a source of Tax, and the LTR-1-Luc reporter revealed no significant difference in LTR-directed gene expression (Fig. 2a). Moreover, cells transfected with either HTLV-1∆CTCF or HTLV-1p12Stop mutant proviral clones produced levels of p19 Gag in the culture supernatant similar to wild-type HTLV-1 (Fig. 2b). Taken together, these data indicate that the inability of the vCTCF-BS to interact with CTCF or the 23 amino acid deletion in p12 have no effect on viral transcription in vitro.

### HTLV-1∆CTCF virus immortalizes primary T-lymphocytes

**Fig. 3:**
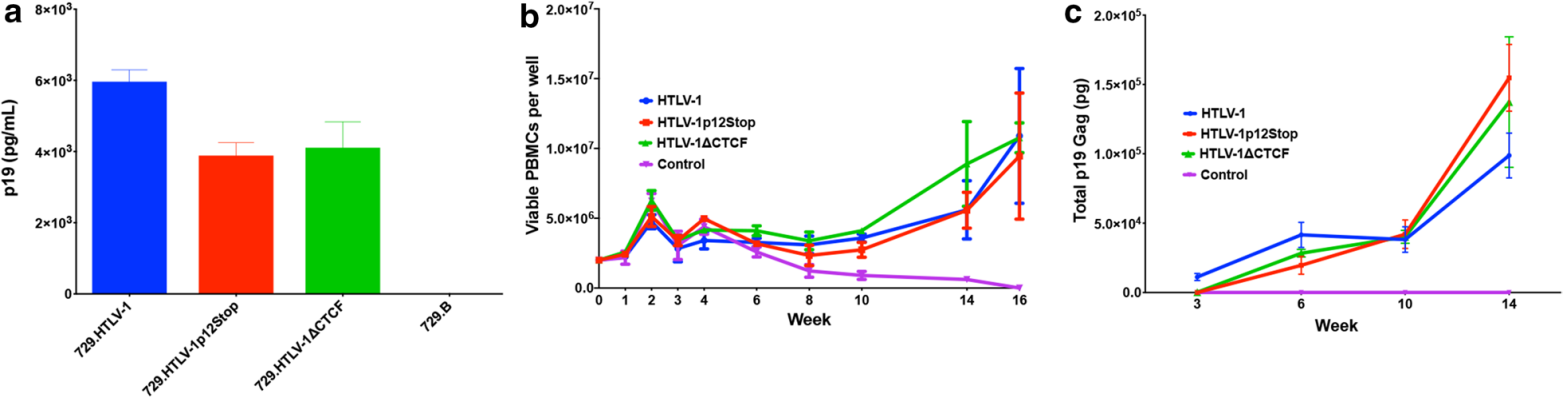
HTLV-1∆CTCF virus immortalizes primary T-lymphocytes. 729 HTLV-1 producer cell clones were generated by nucleofection of 729.B cells with 2 ug of HTLV-1, HTLV-1∆CTCF, HTLV-1p12Stop proviral plasmid clones followed by stable cell selection via G418 treatment and subsequent limiting dilution single cell cloning. 729 HTLV-1 producer cell clones were then irradiated and functionally assessed via p19 Gag ELISA. **a** p19 Gag production was comparable between HTLV-1, HTLV-1∆CTCF, HTLV-1p12Stop producer cell clones. Irradiated producer cell clones (10^6^) were then cocultured in 24-well plates with freshly isolated hPBMCs (2 × 10^6^) to assess in vitro hPBMC immortalization capacity. **b** Viable cells were counted at weeks 0, 1, 2, 3, 4, 6, 8, 10, 14, and 16. HTLV-1, HTLV-1p12Stop, and HTLV-1∆CTCF all maintained hPBMC immortalization capacity. **c** Supernatant was collected and p19 Gag production was measured at weeks 3, 6, 10, and 14. HTLV-1, HTLV-1p12Stop, and HTLV-1∆CTCF displayed comparable p19 Gag production. For figures B and C, the mean (symbols) and standard deviation (error bars) was determined from three random, independent samples from each time point

To determine the capacity of HTLV-1∆CTCF or HTLV-1p12Stop mutant proviral clones to synthesize viral proteins, direct viral replication, and induce cellular immortalization, stable 729 cell transfectants expressing the proviral clones were isolated and characterized. Each stable transfectant contained complete copies of the provirus with the expected mutations (data not shown). To monitor the virion production in these mutant stable transfectants, the concentration of p19 Gag in the culture supernatant of isolated cell clones was quantified by ELISA revealing similar levels of virion production irrespective of wild-type or mutant virus produced (Fig. 3a).

We next assessed the capacity of the HTLV-1 mutant viruses to immortalize human PBMCs in coculture assays. Freshly isolated human PBMCs cocultured with lethally irradiated 729.HTLV-1, 729.HTLV-1∆CTCF, or 729.HTLV-1p12Stop in the presence of 10 U/ml of human IL-2 showed very similar progressive growth patterns consistent with the HTLV-1 immortalization process (Fig. 3b). PBMCs were also cocultured with parental 729 cells as a negative control, and no growth was observed in this condition. We also detected continuous accumulation of p19 Gag in the culture supernatant indicating viral replication and virion production (Fig. 3c). In an effort to obtain a more quantitative measure of the ability of these viruses to infect and immortalize PBMCs, a fixed number of PBMCs (10^4^) were cocultured with tenfold dilutions of virus-producing cells in a 96-well plate assay. Since this assay is very stringent, slowly growing or non-dividing cells are eliminated very quickly and the percentage of surviving wells is an accurate measure of the immortalization efficiency of viruses; PBMCs cocultured with parental 729 uninfected cells as a negative control results in no growth. Data presented in Table 1 indicate that the number of wells containing proliferating lymphocytes was not different between HTLV-1, HTLV-1p12Stop and HTLV-1∆CTCF. Furthermore, flow cytometry and vCTCF-BS DNA sequence analysis of immortalized hPBMCs revealed the expected CD3 + CD4 + T-lymphocyte phenotype and no mutation reversions, respectively (data not shown). Taken together, our results are consistent with the conclusion that CTCF binding to the vCTCF-BS is not required for efficient infectivity or HTLV-1-mediated immortalization of primary human T-lymphocytes in vitro.

**Table 1:** HTLV-1 quantitative immortalization assay

# of producer cells	Number of wells with immortalized cells (48 wells/assay)		
	HTLV-1	HTLV-1∆CTCF	HTLV-1p12Stop
1	2 (4%)	2 (4%)	0 (0%)
10	9 (19%)	8 (17%)	6 (13%)
100	20 (42%)	14 (29%)	14 (29%)
1000	31 (65%)	27 (56%)	28 (58%)

There is no difference between HTLV-1, HTLV-1 p12Stop, and HTLV-1∆CTCF immortalization capacity. Freshly isolated hPBMCs were cocultured with fixed numbers of lethally irradiated viral producer cells in 96-well plates. The number of wells containing proliferating lymphocytes was enumerated.

### HTLV-1 CTCF binding site is dispensable for early in vivo viral persistence

**Fig. 4:**
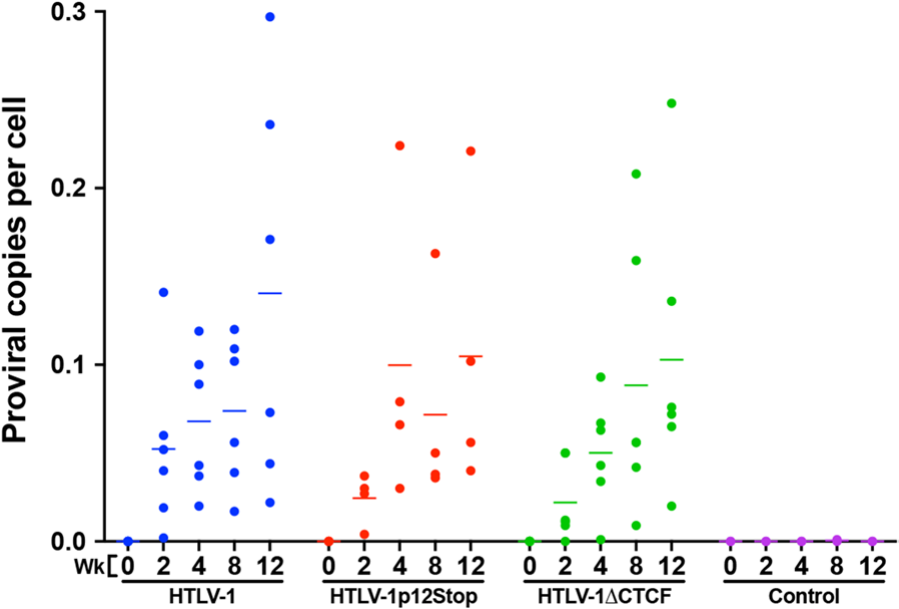
HTLV-1 CTCF binding site is dispensable for early in vivo viral persistence. rPBMC genomic DNA was isolated at 0, 2, 4, 8, and 12 weeks post-infection and subjected to probe-based qPCR using the *Gag/Pol* primer and probe set described in Table 2. Each symbol represents the copy number of a single inoculated rabbit at 0, 2, 4, 8, or 12 weeks post-infection within each group. Bars represent mean copy number per cell. There was no significant difference in proviral load between HTLV-1 or mutant inoculated rabbits. A mixed model analysis with a Bonferroni correction was performed in weeks 8 and 12 to determine statistical significance. A p < 0.0083 was considered a statistically significant change

**Fig. 5:**
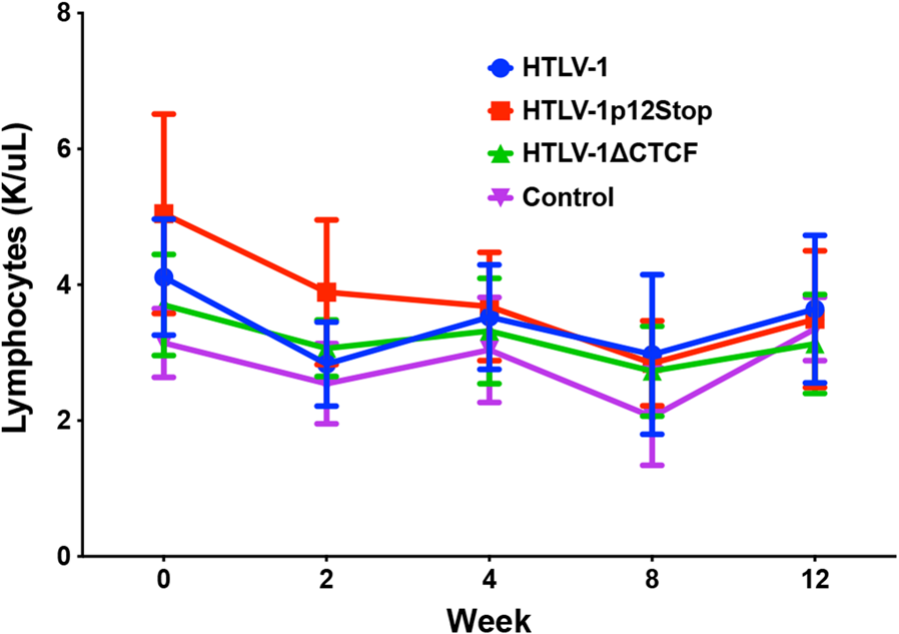
vCTCF-BS ablation confers no change to total lymphocyte count. Manual total lymphocyte count was performed by the OSU Comparative Pathology and Mouse Phenotyping Shared Resource. Symbols represent the mean lymphocyte count and error bars represent standard deviation

To evaluate the role of CTCF in vivo, we compared the abilities of 729, 729.HTLV-1, 729.HTLV-1∆CTCF, or 729.HTLV-1p12Stop cell lines to transmit virus to rabbits, which is an established model of infection and persistence [22, 23]. Rabbits were inoculated with lethally irradiated virus producer cell lines and blood was drawn at select timepoints post-infection (0–12 weeks). Using qPCR, HTLV-1 DNA sequences were detected in the PBMCs of all HTLV-1 and mutant virus-infected rabbits starting as early as week 2 and subsequently throughout the study time course. Quantification of proviral loads revealed variation within individual rabbits, but with a general increase in proviral load over time. There was no significant difference in proviral loads between HTLV-1, HTLV-1∆CTCF, and HTLV-1p12Stop infected rabbits (Fig. 4). Additionally, complete blood count (CBC) results showed no alterations in total lymphocyte count throughout the study (Fig. 5). Diagnostic DNA PCR and nucleotide sequencing performed on PBMCs from rabbits 12 weeks post-infection indicated that the infected cells contained the expected vCTCF-BS and p12 sequences (data not shown). Taken together, our results indicate that CTCF binding is dispensable for efficient HTLV-1 infection, proviral load establishment, and persistence.

### Ablation of HTLV-1 CTCF binding site results in decreased HTLV-1-specific antibody response in infected rabbits

**Fig. 6:**
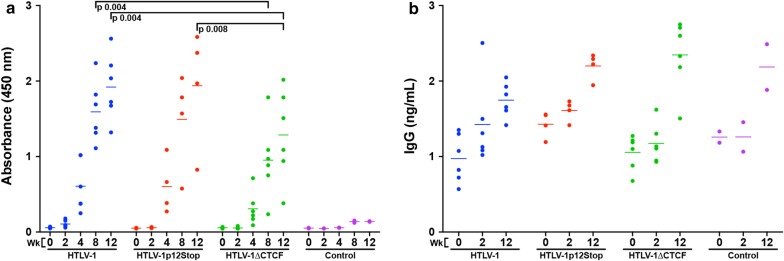
Ablation of HTLV-1 CTCF-binding site significantly decreases HTLV-1-specific antibody response, but not total rabbit IgG. **a** Antibody response was quantified using a modified Avioq HTLV-1/2 Microelisa System protocol (Avioq, Inc., Research Triangle Park, NC). The supplied horseradish peroxidase (HRP) conjugated goat anti-human immunoglobulin (Ig) was substituted for an HRP conjugated goat anti-rabbit IgG (ab6721; Abcam, Cambridge, United Kingdom). Rabbit plasma was diluted 1:500 to obtain absorbance values within the linear range of the assay. Each symbol represents the absorbance value of a single inoculated rabbit at 0, 2, 4, 8, or 12 weeks post-infection within each group. **b** Total rabbit IgG was quantified using the Abcam Rabbit IgG ELISA Kit in accordance with the provided protocol (ab187400; Abcam, Cambridge, United Kingdom). Plasma samples were diluted 1:1 × 10^6^. Each symbol represents total IgG of a single inoculated rabbit at 0, 2, or 12 weeks post-infection within each group. Bars represent mean absorbance or IgG values. Mixed model analyses with a Bonferroni correction were performed in weeks 8 and 12 (HTLV-1-specific) or 2 and 12 (total rabbit IgG) to determine statistical significance. A p < 0.0083 was considered a statistically significant change. Significant changes are denoted by a black line

An important parameter to HTLV-1 infection in vivo is the immune response, particularly specific antibody response to the virus. Plasma was isolated from whole blood samples throughout the study and subjected to a qualitative immunoblot assay and/or quantitative HTLV-specific ELISA. Qualitatively, there appeared to be no significant difference in HTLV-1-specific antibody response throughout the course of the study (Additional file 1: Fig. S1). Quantitatively, HTLV-1, HTLV-1p12Stop, and HTLV-1∆CTCF infected rabbits began to show antibody response at approximately 2–3 weeks post inoculation (Fig. 6a). As previously reported, individual HTLV-1 infected rabbit antibody responses were variable and increased over time [20]. Starting at week 4, it became apparent that antibody response was depressed in HTLV-1∆CTCF infected rabbits compared to HTLV-1 and HTLV-1p12Stop. This depressed antibody response reached statistical significance when compared to HTLV-1 (p 0.004) in week 8 and when compared to HTLV-1 (p 0.004) and HTLV-1p12Stop (p 0.008) at week 12.

To determine the contribution of global changes in antibody response to the changes seen in the HTLV-1-specific antibody response, total rabbit IgG was quantified at weeks 0, 2, and 12 via ELISA (Fig. 6b). Like the HTLV-1-specific antibody response, total rabbit IgG levels rose throughout the study, but no significant difference was detected between HTLV-1∆CTCF and HTLV-1p12Stop and HTLV-1. This suggests that the decrease in HTLV-1-specific antibody response was not the result of significant global alterations in IgG production.

**Fig. 7:**
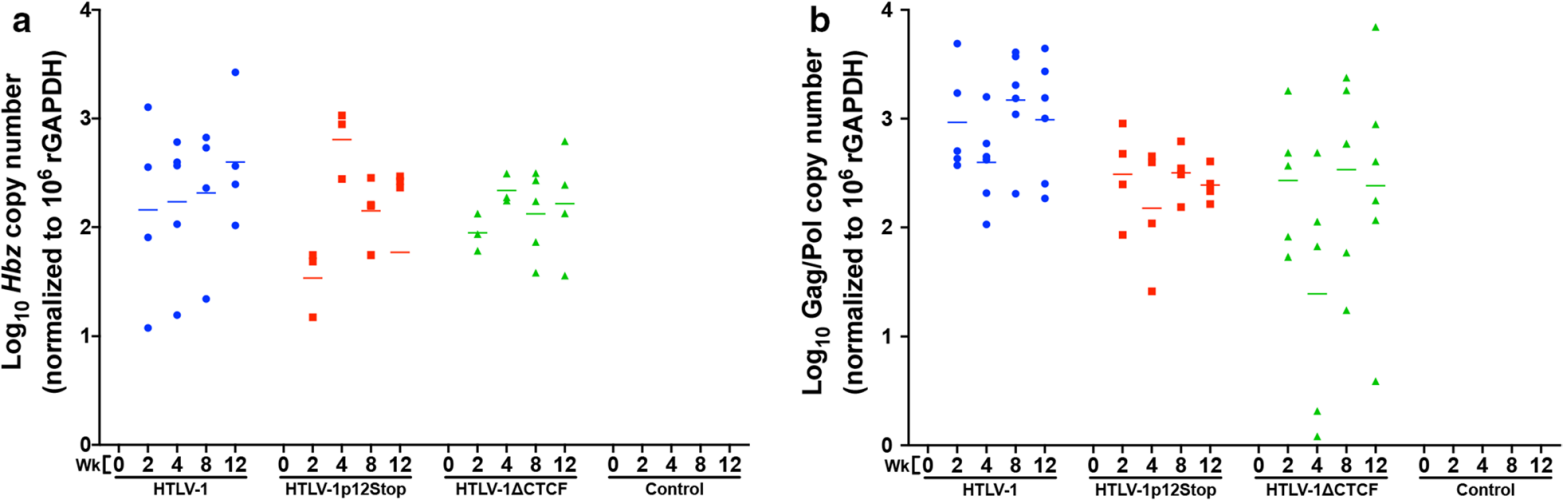
*Hbz* and *Gag/Pol* gene expression is maintained in rabbits infected with HTLV-1∆CTCF. **a** *Hbz* and **b** *Gag/Pol* gene expression was assessed via qPCR. RNA was isolated from rPBMCs, 250 ng of RNA was reverse transcribed, then a 12-cycle cDNA pre-amplification of 2 µL of cDNA was performed followed by a 45-cycle qPCR using 2 µL of pre-amplified cDNA with *Hbz*- or *Gag/Pol*-specific primer and probe sets (Table 2). Total copy number was determined using a standard curve generated by duplicate samples of log^10^ dilutions of *Hbz* or *Gag/Pol* standards listed in Table 2. Copy numbers were normalized to 1 × 10^6^ rGAPDH. There was no significant difference in *Hbz* or *Gag/Pol* gene expression. Each symbol represents the *Hbz* or *Gag/Pol* copy number of a single inoculated rabbit at 0, 2, 4, 8, or 12 weeks post-infection within each group. Bars represent mean *Hbz* or *Gag* copy numbers. Rabbits with a copy number of zero are not represented due to log transformation. *Hbz* gene expression values for two rabbits (week 0 HTLV-1 and week 12 HTLV-1p12Stop) were negative after log transformation and are not graphically represented. A mixed model analysis with a Bonferroni correction was performed in weeks 8 and 12 to determine statistical significance. A p < 0.0083 was considered a statistically significant change

Given the aforementioned reduction in HTLV-1-specific antibody response and the fact that CTCF plays a major role in organization of higher-order chromatin structure and gene expression, we assessed whether loss of CTCF binding in the HTLV-1 proviral genome had an effect on viral gene expression in the infected rabbits over time. RNA was isolated from rPBMCs collected weeks 0–12. RNAs were reverse transcribed, pre-amplified, quantified via qPCR using appropriate primer and probe sets and normalized to 1 × 10^6^ rGAPDH copies. The gene expression analysis was focused on *Hbz*, given its importance in HTLV-1 pathogenesis and its previous consistent expression in the rabbit model of infection [24]. As previously reported, the *Hbz* expression levels are variable in individual animals and steadily rise throughout the study, directly correlating with proviral loads (Fig. 7a) [24]; *Tax* expression spikes as early as 1–2 weeks, is highly variable, and over time becomes low and at the limit of detection [24] (data not shown). Satou et al. recently reported that knockdown of CTCF in vitro resulted in significant repression of *p30* gene expression potentially translating to viral transcriptional changes in infected rabbits [6]. A previous study from our laboratory investigating HTLV-1 gene expression kinetics found *p30* expression to be below the level of detection in the PBMCs of HTLV-1 infected rabbits [24] and analysis of *p30* expression in this study revealed the same (data not shown). However, in an attempt to recapitulate the findings of Satou et al., HTLV-1, HTLV-1p12Stop, and HTLV-1∆CTCF immortalized human peripheral blood leukocytes (immortalized PBLs derived from the coculture assays described in Fig. 3b) were assessed for *p30* gene expression (Additional file 2: Fig. S2). PBL.HTLV-1∆CTCF showed a significant decrease in *p30* gene expression when compared to PBL.HTLV-1p12Stop (p 0.025). While subjectively decreased, the difference in *p30* expression between PBL.HTLV-1∆CTCF and PBL.HTLV-1 was not significant (p 0.175). The statistically significant and subjective changes in *p30* expression are supportive of the previous report by Satou et al. Moreover, *Gag/Pol* expression was utilized as another measure of sense transcription. *Gag/Pol* expression was detectable at week 2 and maintained a steady state throughout the study (Fig. 7b). Statistical analysis revealed no significant differences in *Hbz or Gag/Pol* gene expression between HTLV-1, HTLV-1p12Stop, and HTLV-1∆CTCF infected rabbits (Fig. 7). Despite a lack of statistical significance, *Gag/Pol* gene expression appeared subjectively lower, with higher variability in the HTLV-1∆CTCF infected rabbits when compared to HTLV-1 infected rabbits. A Pearson correlation was performed between the HTLV-1-specific antibody response and *Gag/Pol* gene expression for HTLV-1 (Additional file 3: Fig. S3a), HTLV-1p12Stop (Additional file 3: Fig. S3b), and HTLV-1∆CTCF (Additional file 3: Fig. S3c) at weeks 4, 8, and 12 post-infection. A statistically significant correlation (p < 0.05) was not found at any time point, but HTLV∆CTCF showed a strong positive correlation between HTLV-1-specific antibody response and *Gag/Pol* gene expression at weeks 8 and 12. Comparatively, HTLV-1 and HTLV-1p12Stop had weakly positive to negative correlations at weeks 8 and 12. While not statistically significant, this finding may suggest that the decrease in HTLV-1-specific antibody response for HTLV-1∆CTCF at week 12 may be the result of decreased *Gag/Pol* gene expression.

## Discussion

The HTLV-1 vCTCF-BS was only recently identified, and its role in HTLV-1 replication and/or pathogenesis remains to be defined [6]. Research to date on the vCTCF-BS, has largely been performed in vitro using either ATL-derived cell lines or those derived from chronically infected, asymptomatic infected individuals. These studies have provided valuable insight concerning the effects of the vCTCF-BS on chromatin conformation, proviral and flanking host gene expression, and proviral epigenetic modification [6, 15, 25]. The present study focused on the effects of the CTCF binding site on HTLV-1 in vitro immortalization capacity and early in vivo measures of HTLV-1 viral persistence. We determined that abrogation of the vCTCF-BS had no effect on in vitro immortalization capacity or on in vivo parameters including proviral load, *Hbz or Gag/Pol* gene expression, and viral persistence. However, the loss of the CTCF-binding site did significantly decrease the in vivo HTLV-1-specific antibody response as compared to infected controls (HTLV-1 and HTLV-1p12Stop).

Our in vitro LTR-transactivation data and short-term proliferation and immortalization assays indicated that loss of CTCF binding site was not sufficient to disrupt the capacity of the virus to infect, transcribe, induce proliferation, and/or immortalize primary T-lymphocytes. Therefore, similar to the phenotypes of HTLV-1 open reading frame (ORF) I, II and Hbz protein deletions [20, 26, 27], the CTCF binding site is dispensable for efficient viral infectivity, replication, and primary T-lymphocyte immortalization capacity in vitro.

HTLV-1∆CTCF and HTLV-1p12Stop inoculated rabbits became efficiently infected with HTLV-1. Viral gene expression (as measured by *Hbz* and *Gag/Pol*) and proviral loads were variable in individual rabbits with no significant difference between HTLV-1, HTLV-1p12Stop or HTLV-1∆CTCF persistently infected rabbits. Despite the level of variability, gene expression data displayed trends concurrent with a previous work that examined HTLV-1 gene expression kinetics during early infection of rabbits; *Hbz* expression increased over time with proviral load [24]. A recent study investigated the effects of CTCF-binding on HTLV-1 transcriptional regulation and epigenetic modification in patient-derived PBMCs and HTLV-1-infected T cell clones. The study utilized the CRISPR/Cas9 system to abrogate the vCTCF-BS. Through the use of single-molecule RNA-FISH targeting HTLV-1 transcripts, chromatin immunoprecipitation, and methylated DNA immunoprecipitation the study found that CTCF-binding does not significantly impact viral transcription or epigenetic modification [25]. Thus, this in vitro study is consistent with the result found in our current study in that HTLV-1 CTCF-binding site does not alter HTLV-1 gene expression as measured by LTR transactivation in vitro and *Hbz* and *Gag/Pol* transcript levels in vivo. It is important to note that at several time points during the in vivo study, proviral load and gene expression values in HTLV-1p12Stop infected rabbits were subjectively lower than those in rabbits infected with HTLV-1. A previous study utilized both the NZW rabbit and macaque models of HTLV-1 persistence to investigate the effects of *p12* on infectivity [21]. Rabbits inoculated with a *p12* knockout proviral clone (12KO; first ATG to GTG) showed no decrease in HTLV-1 viral persistence. In the current study, *p12* was truncated by 23 amino acids, as opposed to the previously described complete knockout. While unlikely, these differences in *p12* manipulation could have contributed to in vivo alterations in proviral load and gene expression.

Based on the efficient infectivity and immortalization of T-lymphocytes in vitro and our findings that HTLV-1∆CTCF- and HTLV-1p12Stop-inoculated rabbits became efficiently infected with similar viral gene expression and proviral loads, we were surprised that the HTLV-1-specific antibody response in HTLV-1∆CTCF infected rabbits was significantly decreased. Previous in vivo studies utilizing the NZW rabbit as a model of HTLV viral persistence found that antibody response and proviral load increase in a staggered or tandem fashion [20]. One of those studies examined the effects of Hbz on viral persistence during early infection and found a significant decrease in both antibody response and proviral load when compared to HTLV-1 at later time points [20]. In contrast, another study, examining the effects of HTLV-2 antisense transcript APH-2 under similar in vivo conditions, found an early increase in proviral load followed by a late increase in antibody response [28]. Regardless of the timing, previous studies suggest that a change in proviral load directly correlates with a change in antibody response. In the current study, mean proviral load for HTLV-1∆CTCF infected rabbits was lower than that of HTLV-1, but did not reach significance. It is possible that a significant decrease in proviral load may have developed if the study were allowed to continue past 12 weeks.

The mechanism through which ablation of the vCTCF-BS resulted in a decreased HTLV-1-specific antibody response remains unclear. Several studies have demonstrated a role for CTCF in immune response. CTCF is enriched at antigen receptor loci and has been shown to play a role in chromatin loop organization in these regions [29]. A separate study documented the role of CTCF binding elements (CBEs) in transcriptional regulation and recombination of T-cell and B-cell V(D)J regions. Most recently, Chen et al. utilized DNA adenine methyltransferase identification to identify a lamina-associated domain (LAD) border containing multiple CBEs [30]. Deletion of the aforementioned LAD border resulted in altered T-cell receptor beta locus V(D)J transcription and recombination. Additionally, a prior study found CTCF-mediated alteration of *Tcrd* locus recombination [31]. Similarly, CTCF-mediated alterations of B-cell *Igk* and *Igh* loci recombination have been documented [32, 33]. Adaptive immunity relies on the expression of a diversity of antigen receptors by both T- and B-cells, therefore it is plausible that alteration of CTCF binding, an element known to alter V(D)J recombination, could result in changes to an HTLV-1 specific antibody response. Integration of the vCTCF-BS into the host cell genome could result in altered transcriptional regulation and recombination of T-cell and B-cell V(D)J regions, potentially resulting in increases in adaptive immunity. Deletion of the vCTCF-BS from HTLV-1 clearly decreases the antibody response against HTLV, demonstrating the importance of the vCTCF-BS in eliciting an antibody response. Further studies will be required to dissect how disruption of the vCTCF-BS of HTLV-1 translates to a reduced antibody response.

## Conclusions

The mechanism through which abrogation of CTCF binding alters antibody response in the absence of significant proviral load changes remains unclear. While vCTCF-BS binding is dispensable for early viral replication in vivo, integration site-dependent CTCF interactions may alter proviral load or act as a driving force in tumorigenesis over the course of chronic infection. Studies are underway to investigate the effects of vCTCF-BS in an HTLV-1-induced tumorigenesis mouse model.

## Methods

### Cell culture

Human embryonic kidney (HEK) 293T cells and 729. B cells were cultured in Dulbecco’s modified Eagle medium (DMEM) and Ivscoe’s DMEM, respectively. The culture media was supplemented with 10% fetal bovine serum (FBS), penicillin (100 U/mL), streptomycin (100 μg/mL), and 2 mM glutamine. hPBMCs and rPBMCs were isolated from freshly-collected whole blood using the Ficoll-Paque™ PLUS or Ficoll-Paque™ PREMIUM (GE Healthcare Bio-Sciences AB, Uppsala, Sweden) density gradient medium, respectively. Isolated hPBMCs were cultured in RPMI 1640 medium and supplemented with 20% FBS, 10 U/mL of recombinant human interleukin-2 (IL-2; Roche Diagnostics GmbH, Mannheim, Germany), glutamine, and antimicrobials as described above. All cells were cultured at 37 °C in a humidified atmosphere of 5% CO_2_ and air. Human blood collection protocols were approved by The Ohio State University Office of Responsible Research Practices Institutional Review Board.

### Plasmids and cloning

The infectious HTLV-1 proviral clone contains the Neo^R^ gene and has been previously described [34, 35]. Site-directed mutagenesis of HTLV-1 was used to generate HTLV-1∆CTCF and HTLV-1p12Stop molecular clones. HTLV-1∆CTCF contains several point mutations within the consensus vCTCF-BS while avoiding introduction of mutations to the opposite-strand coding sequence of the *Hbz* gene. However, the vCTCF-BS mutations do result in changes to *p12*, a sense transcribed HTLV-1 accessory gene. In lieu of producing a *p12* gene product with multiple substitutions and potentially confounding results, an additional mutation was introduced in *p12*, immediately upstream of the vCTCF-BS mutations that results in deletion of the carboxy terminal 23 amino acids of p12 (Fig. 1a). HTLV-1p12Stop contains only the p12 stop point mutations, and thus served as a control for potential effects of the p12 deletion in our viral studies. Ablation of CTCF binding to the HTLV-1∆CTCF molecular clone was confirmed via EMSA.

EMSA was performed by using Light Chemiluminescent EMSA kit (Thermo Scientific) and following the manufacturer’s protocol with some modification. Briefly nuclear extract of 293T cells transfected with the plasmid overexpressing human CTCF protein was incubated with biotin labeled target DNA in 1 × binding buffer containing 5 mM MgCL_2_, 25 μM ZnSO_4_, 2.5% glycerol, 50 ng/ml poly(dI-dC) and proteinase inhibitor cocktail in the presence and absence of the CTCF antibody for 30 min at room temperature. Protein bound DNA was separated from unbound DNA in a 6% polyacrylamide gel in TBE buffer and transferred to a nylon membrane. DNA was then cross-linked to the membrane with the UV Stratalinker 1800 (Stratagene) for 1 min. After 15 min blocking, the membrane was incubated with streptavidin–horseradish peroxidase conjugate in blocking buffer for 15 min and then exposed to the substrate solution. Biotin labeled DNA was detected by using Chemidoc XRS + molecular imager (Bio-Rad). The sequences of target DNA oligos: (1) DNA with wild type CTCF binding site: ATGCGTTTCCCCGCGAGGTGGCGCTTTCTCCCC. (2) DNA with mutated CTCF binding site: ATGCGTTTCCCCTCTCGGTGGAGCTTTCTCCCC. The LTR-1-Luc and thymidine kinase (TK)-*Renilla* reporter plasmids were described previously [36].

### In vitro HTLV-1 functional assays

HEK293T cells were transfected using Mirus TransIT^®^-2020 transfection reagent (Mirus Bio LLC, Madison, WI) according to manufacturer’s instructions. HEK293T cells were co-transfected with 1 μg of HTLV-1, HTLV-1∆CTCF, HTLV-1 p12Stop, or empty (pcDNA3) plasmids along with 100 ng of LTR-1-Luc and 20 ng of TK-*Renilla* reporter plasmids. An HTLV-1 p19 Gag enzyme-linked immunosorbent assay (ELISA; Zeptometrix Corporation, Buffalo, NY) was performed with supernatant collected 48 h post-transfection. Transfected cells were also harvested at the time of supernatant collection. Cell pellets were lysed and HTLV-1 LTR-transactivation was measured via luciferase assay according to the manufacturer’s protocol (Dual-Luciferase^®^ Reporter Assay System, Promega Corporation, Madison, WI; Filtermax F5 Multi-Mode Microplate Reader, Molecular Devices, San Jose, CA) [35]. Assays were performed with LTR-1-luc activity normalized for transfection efficiency using *Renilla* luciferase.

### Producer cell generation

Stable 729 HTLV-1 producer cell clones were generated by nucleofection of 729.B cells with 2 µg of HTLV-1, HTLV-1∆CTCF, or HTLV-1p12Stop plasmid using an Amaxa Cell Line Nucleofector™ Kit V in accordance with the manufacturer’s suggested protocols (program X-001; Amaxa, Cologne, Germany). Nucleofected cells were then subjected to G418 selection (1 mg/mL; Life Technologies, Carlsbad, CA). An HTLV-1 ELISA was used to confirm p19 Gag production in G418 selected cell lines. Cell lines with p19 Gag production were then single cell selected via limiting dilution. HTLV-1∆CTCF and HTLV-1 p12Stop mutations were confirmed via Sanger sequencing (see Methods: PCR and quantitative PCR). p19 Gag ELISAs were performed on single cell clones and those with comparable p19 Gag production were selected for coculture immortalization assays.

### Coculture immortalization assay

1 × 10^6^ 729. B cells and 729 HTLV-1 producer cell clones (HTLV-1, HTLV-1∆CTCF and HTLV-1 p12Stop) were lethally irradiated (100 Gy) and cocultured with freshly-isolated hPBMCs (2 × 10^6^) in the presence of IL-2 (10 U/mL, replenished once weekly). Three randomly selected wells from each coculture were counted weekly via trypan blue exclusion and p19 Gag concentration was measured by ELISA at weeks 3, 6, 10, and 14. Wells with continuous hPBMC expansion in conjunction with p19 Gag expression in the presence of IL-2 were scored as immortalized. HTLV-1∆CTCF and HTLV-1p12Stop immortalized hPBMCs were checked for reversions via Sanger sequencing (see “Methods”: PCR and quantitative PCR). To quantify the immortalization ability of HTLV-1 mutant viruses we performed an immortalization assay using human PBMC. Freshly isolated hPBMCs (10^4^ cells/well) were co cultured with tenfold dilutions (10^3^, 10^2^, 10^1^, 1) of lethally irradiated 729.HTLV-1, 729.HTLV-1∆CTCF, or 729.HTLV-1p12Stop in the presence of 10 U/ml of human IL-2 in a 96 well plate (48 replicates each). The number of wells which became immortalized were determined by microscopy.

### In vivo HTLV-1 infection

After a 2 week acclimatization period, fourteen-week-old, intact, male, specific pathogen-free New Zealand White rabbits (Crl:KBL (NZW); Charles River Laboratories, Wilmington, MA) were inoculated via the lateral ear vein with 1 × 10^7^ lethally irradiated (100 Gy) 729 producer cell clones or 729.B control cells. A portion of lethally irradiated cells (1 × 10^6^) were maintained in cell culture to assess p19 Gag production 24 h post-irradiation and ensure cell death. Blood was drawn via the central auricular artery pre-infection (week 0) and at weeks 2, 4, 8, and 12 post-infection. rPBMCs and plasma were isolated from freshly-collected blood using Ficoll-Paque™ PREMIUM (GE Healthcare Bio-Sciences AB, Uppsala, Sweden) density gradient medium. Whole blood samples were analyzed for CBC at each time point (The Ohio State University Comparative Pathology and Mouse Phenotyping Shared Resource, Columbus, OH). rPBMCs or plasma were assessed for proviral load, HTLV-1 gene expression, and HTLV-1-specific antibody response, as described below. Sanger sequencing of the vCTCF-BS was performed at week 12 to monitor for viral reversions. All animal procedures were performed in accordance with a protocol approved by University Laboratory Animal Resources (ULAR) of The Ohio State University.

### PCR and quantitative PCR

DNA isolation from 729 HTLV-1 producer cell clones and coculture-immortalized hPBMCs was performed using the Qiagen DNeasy Kit (Qiagen, Valencia, CA). Standard PCR followed by Sanger sequencing for vCTCF-BS mutation verification was performed for each newly-generated producer cell clone and coculture-immortalized hPBMCs (hPBMCs collected at week 16 of coculture assay). vCTCF-BS primer sets (Table 2) and the following PCR conditions were utilized for PCR amplification: 95°C for 3 min followed by 35 cycles of 95°C for 15 s, 60°C for 1 min. Amplified PCR product for each sample was then purified using the QIAquick PCR Purificaiton Kit and submitted for Sanger sequencing (Qiagen, Valencia, CA). Sequencing was performed with individual reactions for the forward and reverse vCTCF-BS primers (Table 2).

**Table 2:** Primers and probes used for HTLV-1 Gene expression, proviral load, and PCR for sequencing

mRNA	Plasmid standard	Primers and probe	
*Hbz*	JA662	5′ Primer	[HBZMAP1] 5′-^1905^CTTCTAAGGATAGCAAACCGTCAAG^1881^-3′
		Probe	[TMP-13] 5′-/56-FAM/^1782^CCTGTGCCA/ZEN/TGCCCGGAGGA^1801^/3IABkFQ/-3′
		3′ Primer	[HBZMAP2] 5′-^353^ATGGCGGCCTCAG^365^1765^GGCT^1768^-3′
*rGAPDH*	rGAPDH	5′ Primer	[rGAPDH-S] 5′-GATGCTGGTGCCGAGTACGTG-3′
		Probe	[BY-1Z] 5′-/56-FAM/ACCACCATG/ZEN/GAGAAGGCCGGG/3IABkFQ/-3′
		3′ Primer	[rGAPDH-AS] 5′- GTGGTGCAGGATGCGTTGCTGA-3′
*Gag/Pol*	ACHneo	5′ Primer	[#20] 5′-^938^AGCCCCCAGTTCATGCAGACC^958^-3′
		Probe	[TMP-3] 5′-/56-FAM/^990^CTGCCAAAG/ZEN/ACCTCCAAGACCTCC^1013^/3IABkFQ/-3′
		3′ Primer	[#19] 5′-^1036^GAGGGAGGAGCAAAGGTACTG^1016^-3′
Gene			
*Gag/Pol*	ACHneo	5′ Primer	[#20] 5′-^938^AGCCCCCAGTTCATGCAGACC^958^-3′
		Probe	[TMP-3] 5′-/56-FAM/^990^CTGCCAAAG/ZEN/ACCTCCAAGACCTCC^1013^/3IABkFQ/-3′
		3′ Primer	[#19] 5′-^1036^GAGGGAGGAGCAAAGGTACTG^1016^-3′

Region of interest	Plasmid standard	Primers	
vCTCF-BS	NA	5′ Primer	[CTCF-F2] 5′-^6826^TAGCACTATGCTGTTTCGCCT^6846^-3′
		3′ Primer	[CTCF-R2] 5′-^7211^GGTGGACGGGCTATTATCTT^7192^-3′

All provided sequence numbering is in the context of the HTLV-1 molecular clone ACHneo

DNA and RNA were isolated from rPBMCs using the AllPrep DNA/RNA Mini Kit (Qiagen, Valencia, CA). Proviral load was quantitated with probe-based qPCR using 250 ng of rPBMC gDNA, Bio-Rad iQ™ Supermix, and the *Gag/Pol* primer/probe set described in Table 2 (Bio-Rad Laboratories, Hercules, CA; Integrated DNA Technologies, Coralville, IA). The qPCR conditions were as follows: 94 °C for 3 min followed by 45 cycles of 94 °C for 15 s, 55 °C for 30 s, 72 °C for 40 s. Total copy number was determined using a standard curve generated by duplicate samples of log^10^ dilutions of the ACHneo plasmid. Copy number per cell was generated based on the estimation that 1 μg of rPBMC DNA is equivalent to 134,600 cells, as previously described [37]. DNA isolated from PBMCs from each rabbit (excluding uninfected 729B control rabbits) at week 12 of the study was screened individually for mutation reversion utilizing standard PCR amplification followed by submission for Sanger sequencing. vCTCF-BS primer sets (Table 2) and the following PCR conditions were utilized for PCR amplification: 95 °C for 3 min followed by 35 cycles of 95 °C for 15 s, 60 °C for 1 min. Amplified PCR product for each rabbit was then purified using the QIAquick PCR Purification Kit and submitted for Sanger sequencing (Qiagen, Valencia, CA). Sequencing was performed with individual reactions for the forward and reverse vCTCF-BS primers (Table 2).

*Hbz* and *Gag/Pol* gene expression was quantified via cDNA synthesis followed by a pre-amplification and qPCR. cDNA synthesis was performed using the SuperScript™ IV First-Strand Synthesis System with 250 ng rPBMC RNA and appropriate-RT controls (Invitrogen, Carlsbad, CA). Pre-amplification was performed using the SsoAdvanced™ PreAmp Supermix with 2 μL of rPBMC cDNA. The pre-amplification pool included primers for *Hbz, Gag/Pol,* and *rGAPDH* in accordance with the manufacturer’s protocol (primers listed in Table 2). The pre-amplification conditions were as follows: 95 °C for 3 min followed by twelve cycles of 95 °C for 15 s, 58 °C for 4 min. Pre-amplified products were diluted 1:5 per the manufacturer’s protocol. Given the abundance of rGAPDH compared to the gene of interest (*Hbz*), pre-amplified products destined for rGAPDH qPCR were diluted 1:50 in order to obtain acceptable Ct values. A 45-cycle qPCR was performed in duplicate with appropriate minus-RT controls using Bio-Rad iQ™ Supermix with 2 μL of pre-amplified cDNA and *Hbz* or *rGPADH* primer/probe sets described in Table 2 (Bio-Rad Laboratories, Hercules, CA; Integrated DNA Technologies, Coralville, IA). The qPCR conditions were as follows: 95 °C for 3 min followed by 45 cycles of 95 °C for 15 s, 57.5 °C for 30 s. Total copy number was determined using a standard curve generated by duplicate samples of log^10^ dilutions of the *Hbz* standard listed in Table 2. Copy numbers were normalized to 1 × 10^6^ rGAPDH. A mixed model analysis with a Bonferroni correction was performed in weeks 8 and 12 to determine statistical significance. A p < 0.0083 was considered a statistically significant change.

In vitro *p30* gene expression was quantified via cDNA synthesis followed by qPCR. cDNA synthesis was performed using the SuperScript™ IV First-Strand Synthesis System with 1 µg HTLV-1, HTLV-1∆CTCF, or HTLV-1p12Stop immortalized PBL RNA and appropriate-RT controls (Invitrogen, Carlsbad, CA). A 45-cycle qPCR was performed in duplicate with appropriate minus-RT controls using Bio-Rad iQ™ Supermix with 2 μL of cDNA and *p30* or human *GAPDH (hGPADH)* primer/probe sets (Bio-Rad Laboratories, Hercules, CA; Integrated DNA Technologies, Coralville, IA). *p30* primer/probe set were previously described by Li et al. [24]. *hGAPDH* quantification utilized Integrated DNA Technologies (IDT) PrimeTime^®^ Predesigned qPCR Assay Hs.PT.39a.22214836 in conjunction with a custom IDT gBlock gene fragment; GCGCCGCTGCGGGCCGAGCCACATCGCTCAGACACCATGGGGAAGGTGAAGGTCGGAGTCAACGGATTTGGTCGTATTGGGCGCCTGGTCACCAGGGCTGCTTTTAACTCTGGTAAAGTGGATATTGTTGCCATCAATGACCCCTTCATTGACCTCAACTACATGGTTTACATGTTCCAATAT (Integrated DNA Technologies, Coralville, IA). The qPCR conditions were as follows: 95 °C for 3 min followed by 45 cycles of 95 °C for 15 s, 60 °C for 30 s. Total copy number was determined using a standard curve generated by duplicate samples of log^10^ dilutions of the *p30* standard previously described by Li et al. [24]. Copy numbers were normalized to 1 × 10^6^ *hGAPDH*. One way ANOVA with multiple comparisons was used for statistical analysis with significance denoted by p < 0.05.

A Pearson Correlation was performed between the HTLV-1-specific antibody response and *Gag/Pol* gene expression for HTLV-1, HTLV-1∆CTCF, and HTLV-1p12Stop at weeks 4, 8, and 12 post-infection. A statistically significant correlation is denoted by p < 0.05.

### HTLV-1 antibody response assays

The HTLV antibody response was assessed qualitatively in a representative rabbit from each condition via a modified MP Diagnostics HTLV Blot 2.4 Western Blot Assay protocol (MP Biomedicals LLC, Santa Ana, CA). The supplied alkaline phosphatase conjugated goat anti-human immunoglobulin gamma (IgG) was substituted for an alkaline phosphatase conjugated goat anti-rabbit IgG (ab6722; Abcam, Cambridge, United Kingdom). Plasma from each condition was diluted 1:10.

After qualitative assessment of representative rabbits, HTLV-1-specific antibody response was quantified for all rabbits using a modified Avioq HTLV-1/2 Microelisa System protocol (Avioq, Inc., Research Triangle Park, NC). The supplied horseradish peroxidase (HRP) conjugated goat anti-human IgG was substituted for an HRP conjugated goat anti-rabbit IgG (ab6721; Abcam, Cambridge, United Kingdom). Rabbit plasma was diluted 1:500 to obtain absorbance values within the linear range of the assay.

Total rabbit IgG was quantified using the Abcam Rabbit IgG ELISA Kit in accordance with the provided protocol (ab187400; Abcam, Cambridge, United Kingdom). Plasma samples were diluted 1:1 × 10^6^. Mixed model analyses with a Bonferroni correction were performed in weeks 8 and 12 (HTLV-1-specific) or 2 and 12 (total rabbit IgG) to determine statistical significance. A p < 0.0083 was considered a statistically significant change.

## Supplementary information

**Additional file 1: Fig. S1.** Ablation of HTLV-1 CTCF-binding site does not qualitatively decrease HTLV-1-specific antibody response. The HTLV antibody response was assessed qualitatively at 0, 4, and 12 weeks post-inoculation in a representative rabbit from each condition via a modified MP Diagnostics HTLV Blot 2.4 Western Blot Assay protocol (MP Biomedicals LLC, Santa Ana, CA). The supplied alkaline phosphatase conjugated goat anti-human immunoglobulin gamma (IgG) was substituted for an alkaline phosphatase conjugated goat anti-rabbit IgG (ab6722; Abcam, Cambridge, United Kingdom). Plasma from each condition was diluted 1:10. Reactive HTLV-1 proteins are labeled on the left. rgp46-1 (HTLV-1-specific recombinant envelope surface protein); p53 (Gag precursor); p24 (capsid protein); p19 (matrix protein); GD21 (recombinant transmembrane envelope protein). “Serum control” denotes serum immunoglobulin levels among.

**Additional file 2: Fig. S2.** Ablation of the HTLV-1 CTCF-binding site significantly decreases HTLV-1 *p30* expression in vitro in HTLV-1∆CTCF immortalized PBLs when compared to HTLV-1p12Stop, but not when compared to HTLV-1. *p30* gene expression was assessed via qPCR. RNA was isolated from HTLV-1-immortalized (PBL.HTLV-1), HTLV-1∆CTCF-immortalized (PBL.HTLV-1∆CTCF, and HTLV-1p12Stop-immortalized (PBL.HTLV-1p12Stop) PBLs. cDNA was synthesized from 1 µg of RNA, then a 45-cycle qPCR was performed in duplicate using 2 µL of cDNA per reaction and a *p30*-specific primer/probe set. Total copy number was determined using a standard curve generated by duplicate samples of log^10^ dilutions of *p30* standard (primer/probe set and standard described in materials and methods). Copy numbers were normalized to 10^6^ *human GAPDH* (*hGAPDH*). Bars represent mean log^10^ *p30* copy normalized to *hGAPDH*. Error bars indicated standard deviation. PBL.HTLV-1∆CTCF showed a significant decrease in *p30* gene expression when compared to PBL.HTLV-1p12Stop (p 0.025). While subjectively decreased, the difference in *p30* expression between PBL.HTLV-1∆CTCF and PBL.HTLV-1 was not significant (p 0.175). One way ANOVA with multiple comparisons was used for statistical analysis with significance denoted by values with p < 0.05.

**Additional file 3: Fig. S3.** There is a positive correlation between HTLV-1-specific antibody response and *Gag/Pol* gene expression in HTLV-1∆CTCF-infected rabbits. A Pearson Correlation was performed between the HTLV-1-specific antibody response and *Gag/Pol* gene expression for HTLV-1 (**A**), HTLV-1p12Stop (**B**), and HTLV-1∆CTCF (**c**) at weeks 4, 8, and 12 post-infection. A statistically significant correlation (p < 0.05) was not found at any time point, but HTLV-1∆CTCF showed a strong positive correlation between HTLV-1-specific antibody response and Gag/Pol gene expression at weeks 8 and 12. Comparatively, HTLV-1 and HTLV-1p12Stop had weakly positive to negative correlations at weeks 8 and 12. While not statistically significant, this finding may suggest that the decrease in HTLV-1-specific antibody response for HTLV-1∆CTCF at week 12 may be the result of decreased *Gag/Pol* gene expression.

## Data Availability

The datasets used and/or analyzed during the current study are available from the corresponding author on reasonable request.
