# Supplementary figures and images for: HTLV-1 CTCF-binding site is dispensable for in vitro immortalization and persistent infection in vivo
Source: Retrovirology. 2019 Dec 21;16:44. doi: 10.1186/s12977-019-0507-9 (PMC6925871; doi:10.1186/s12977-019-0507-9)

Serum Control-  
rgp46-1-

p53-

p24-

p19-

GD21-

HTLV-1

HTLV-1p12Stop

HTLV-1 $\Delta$ CTCF

Control

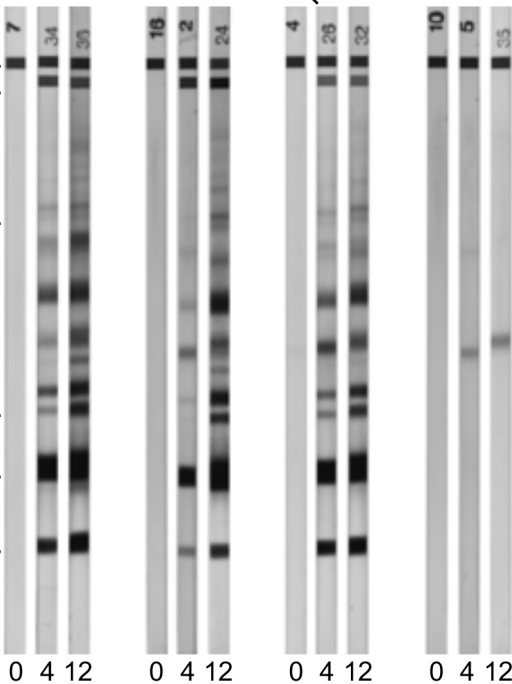

Supplement: Supplementary file 1 — Additional file 1: Fig. S1. Ablation of HTLV-1 CTCF-binding site does not qualitatively decrease HTLV-1-specific antibody response. The HTLV antibody response was assessed qualitatively at 0, 4, and 12 weeks post-inoculation in a representative rabbit from each condition via a modified MP Diagnostics HTLV Blot 2.4 Western Blot Assay protocol (MP Biomedicals LLC, Santa Ana, CA). The supplied alkaline phosphatase conjugated goat anti-human immunoglobulin gamma (IgG) was substituted for an alkaline phosphatase conjugated goat anti-rabbit IgG (ab6722; Abcam, Cambridge, United Kingdom). Plasma from each condition was diluted 1:10. Reactive HTLV-1 proteins are labeled on the left. rgp46-1 (HTLV-1-specific recombinant envelope surface protein); p53 (Gag precursor); p24 (capsid protein); p19 (matrix protein); GD21 (recombinant transmembrane envelope protein). “Serum control” denotes serum immunoglobulin levels among. [file 12977_2019_507_MOESM1_ESM.pdf]

Log<sub>10</sub> p30 copy number  
(normalized to 10<sup>6</sup> hGAPDH)

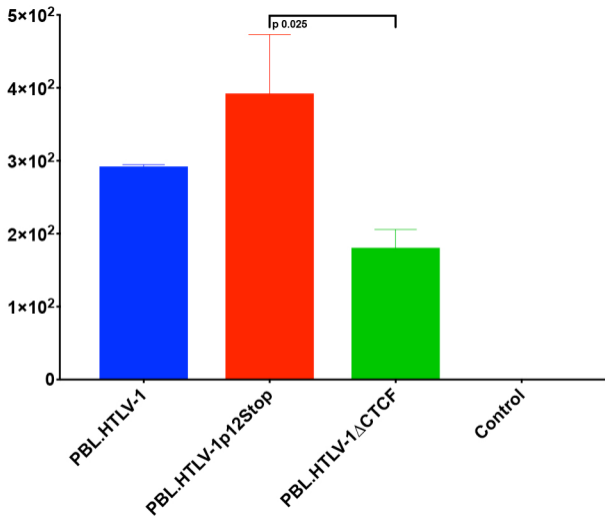

Supplement: Supplementary file 2 — Additional file 2: Fig. S2. Ablation of the HTLV-1 CTCF-binding site significantly decreases HTLV-1 p30 expression in vitro in HTLV-1∆CTCF immortalized PBLs when compared to HTLV-1p12Stop, but not when compared to HTLV-1. p30 gene expression was assessed via qPCR. RNA was isolated from HTLV-1-immortalized (PBL.HTLV-1), HTLV-1∆CTCF-immortalized (PBL.HTLV-1∆CTCF, and HTLV-1p12Stop-immortalized (PBL.HTLV-1p12Stop) PBLs. cDNA was synthesized from 1 µg of RNA, then a 45-cycle qPCR was performed in duplicate using 2 µL of cDNA per reaction and a p30-specific primer/probe set. Total copy number was determined using a standard curve generated by duplicate samples of log10 dilutions of p30 standard (primer/probe set and standard described in materials and methods). Copy numbers were normalized to 106 human GAPDH (hGAPDH). Bars represent mean log10 p30 copy normalized to hGAPDH. Error bars indicated standard deviation. PBL.HTLV-1∆CTCF showed a significant decrease in p30 gene expression when compared to PBL.HTLV-1p12Stop (p 0.025). While subjectively decreased, the difference in p30 expression between PBL.HTLV-1∆CTCF and PBL.HTLV-1 was not significant (p 0.175). One way ANOVA with multiple comparisons was used for statistical analysis with significance denoted by values with p < 0.05. [file 12977_2019_507_MOESM2_ESM.pdf]

A

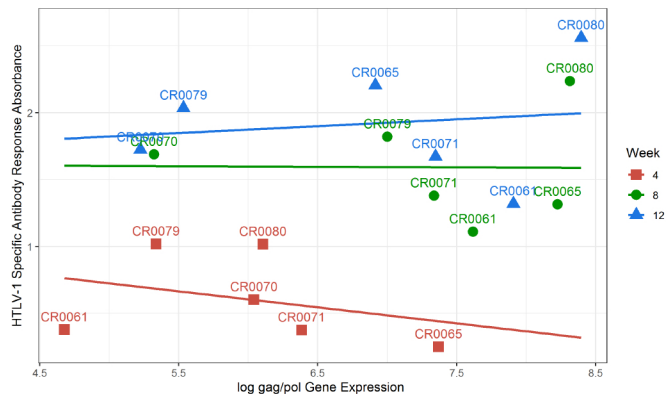

B

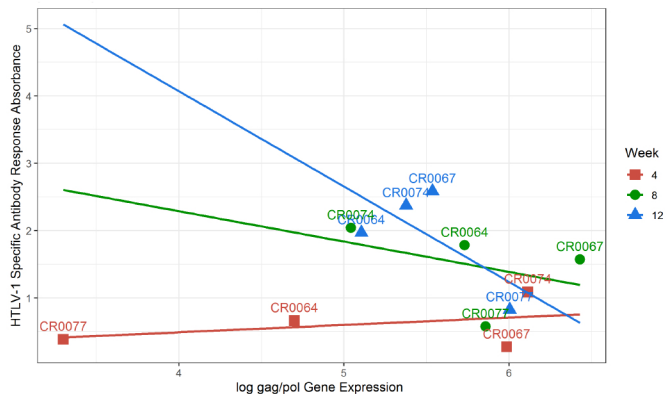

C

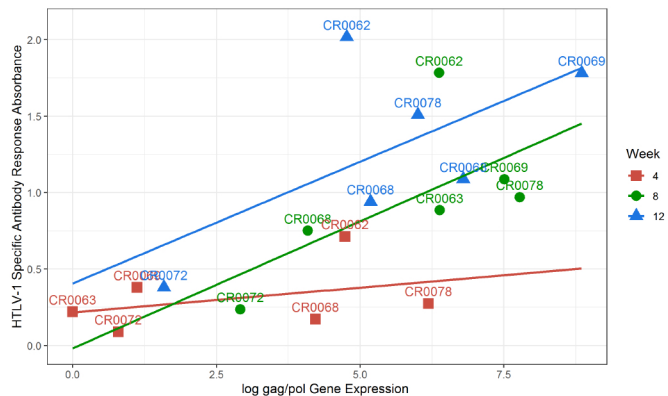

Supplement: Supplementary file 3 — Additional file 3: Fig. S3. There is a positive correlation between HTLV-1-specific antibody response and Gag/Pol gene expression in HTLV-1∆CTCF-infected rabbits. A Pearson Correlation was performed between the HTLV-1-specific antibody response and Gag/Pol gene expression for HTLV-1 (A), HTLV-1p12Stop (B), and HTLV-1∆CTCF (c) at weeks 4, 8, and 12 post-infection. A statistically significant correlation (p < 0.05) was not found at any time point, but HTLV-1∆CTCF showed a strong positive correlation between HTLV-1-specific antibody response and Gag/Pol gene expression at weeks 8 and 12. Comparatively, HTLV-1 and HTLV-1p12Stop had weakly positive to negative correlations at weeks 8 and 12. While not statistically significant, this finding may suggest that the decrease in HTLV-1-specific antibody response for HTLV-1∆CTCF at week 12 may be the result of decreased Gag/Pol gene expression. [file 12977_2019_507_MOESM3_ESM.pdf]
